# Supplementary material for: Gender inequity in speaking opportunities at the American Geophysical Union Fall Meeting
Source: Nat Commun. 2018 Apr 24;9:1358. doi: 10.1038/s41467-018-03809-5 (PMC5915405; doi:10.1038/s41467-018-03809-5)
Supplement: Supplementary file 1 — Supplementary Information [file 41467_2018_3809_MOESM1_ESM.pdf]

Supplementary Materials

Gender inequity in speaking opportunities at the American Geophysical Union Fall Meeting

Ford et al.,

Supplementary Figure 1. The proportion of women in the Sections and invited authors, author assigned oral presentations and authors opting for "poster only" presentations.

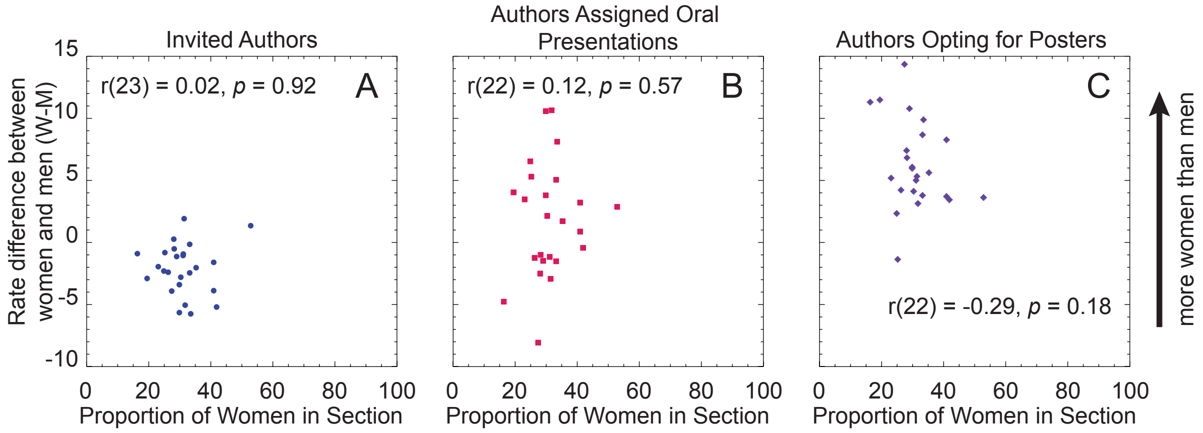

For all Supplementary Tables, significant results ($p < 0.05$) are in **bold**.

Supplementary Table 1. Analysis of Invited Authors by First Author Career Stage

	Total		*Student*		*Early Career*		*Mid-Career*		*Experienced*		*Retired*	
Total Abstracts	65,247		18789		23112		14815		7307		186	
	F	M	F	M	F	M	F	M	F	M	F	M
	20900	44347	8042	10747	7900	15212	3548	11267	1157	6150	18	168
	32%	68%	43%	57%	34%	66%	24%	76%	16%	84%	10%	90%
Invited Authors	2040	5499	124	192	862	1501	725	2134	281	1477	2	22
Invited Authors %	9.8%	12.4%	1.5%	1.8%	10.9%	9.9%	20.4%	18.9%	24.3%	24.0%	11.1%	13.1%
mean	1.10	1.12	1.02	1.02	1.11	1.10	1.20	1.19	1.24	1.24	1.11	1.13
std	0.297	0.330	0.123	0.132	0.312	0.298	0.403	0.392	0.429	0.427	0.323	0.338
$\chi 2$	**96.8**		1.66		**6.18**		**3.87**		0.0391		0.0570	
p-value	**<0.001**		0.197		**0.013**		**0.049**		0.843		0.811	

Supplementary Table 2. Analysis of Oral Presentations by First Author Career Stage

	Total		*Student*		*Early Career*		*Mid-Career*		*Experienced*		*Retired*	
Total Abstracts	31348		7275		11293		7913		4138		85	
	F	M	F	M	F	M	F	M	F	M	F	M
	9424	21924	3017	4258	3880	7413	1808	6105	584	3554	10	75
	30%	70%	41%	59%	34%	66%	23%	77%	14%	86%	12%	88%
Assigned Oral	3874	9759	869	1173	1633	3080	960	3138	340	2064	3	38
Assigned Oral %	41.1%	44.5%	28.8%	27.5%	42.1%	41.5%	53.1%	51.4%	58.2%	58.1%	30.0%	50.7%
mean	1.59	1.55	1.71	1.72	1.58	1.58	1.47	1.49	1.42	1.42	1.70	1.49
std	0.492	0.485	0.453	0.447	0.494	0.493	0.499	0.500	0.494	0.494	0.483	0.503
$\chi 2$	**31.1**		1.38		0.304		1.61		0.00430		1.51	
p-value	**<0.001**		0.24		0.581		0.205		0.948		0.219	

Supplementary Table 3. Analysis of Invited Authors, Authors Assigned Oral Presentation and Authors Opting for “Poster Only” Presentations by Sections. Due to the relatively small numbers within each Section, the absolute numbers are not provided in order to maintain anonymity.

		Rate Difference Between Women and Men (Women - Men)		
Sections	*Proportion of Women*	*Invited Authors*	*Assigned Oral Presentation*	*Authors Opting for “Poster Only” Presentations*
Atmospheric and Space Electricity	*16%*	-0.9%	-4.8%	11.3%
Atmospheric Sciences	*32%*	-5.1%	10.6%	3.1%
Biogeosciences	*41%*	-3.9%	3.2%	3.7%
Cryosphere	*31%*	-0.9%	-1.2%	5.0%
Earth and Planetary Surface Processes	*33%*	-0.1%	-1.5%	3.8%
Earth and Space Science Informatics	*26%*	-2.4%	-1.3%	4.2%
Education	53%	1.3%	2.9%	3.6%
Geodesy	25%	-2.3%	6.5%	2.3%
Geomagnetism, Paleomagnetism and Electromagnetism	27%	-3.9%	-8.1%	14.4%
Global Environmental Change	34%	-5.8%	8.1%	9.9%
Hydrology	30%	-3.4%	3.8%	6.1%
Mineral and Rock Physics	29%	-1.1%	-1.5%	10.8%
Natural Hazards	30%	-5.7%	10.6%	6.0%
Near Surface Geophysics	25%	-0.8%	5.3%	-1.4%
Nonlinear Geophysics	19%	-2.9%	4.0%	11.5%
Ocean Sciences	33%	-2.4%	5.1%	8.7%
Paleoceanography and Paleoclimatology	41%	-1.6%	0.9%	8.3%
Planetary Sciences	28%	0.3%	-2.5%	7.4%
Public Affairs	42%	-5.2%	-0.4%	3.4%
Seismology	28%	-0.5%	-1.0%	6.8%
Space Physics & Aeronomy	23%	-2.0%	3.5%	5.2%
Study of Earth's Deep Interior	31%	1.9%	-2.9%	5.3%
Tectonophysics	30%	-2.8%	2.1%	4.1%
Union	31%	-1.0%	-	-
Volcanology, Geochemistry and Petrology	35%	-2.0%	1.7%	5.6%
	r	0.02	0.12	-0.29
	df	23	22	22
	p-value	0.92	0.57	0.18

Supplementary Table 4. Analysis of Primary Convener Allocation of Invited Authors by First Author Career Stage

	Total		*FA Student*		*FA Early Career*		*FA Mid-Career*		*FA Experienced*		*FA Retired*	
Total Abstracts	7442		314		2336		2818		1733		24	
Female Primary Convener	2081	28%	87	28%	695	30%	790	28%	447	26%	9	38%
	F	M	F	M	F	M	F	M	F	M	F	M
Invited Authors	716	1365	38	49	309	386	258	532	93	354	2	7
Invited Authors %	34%	66%	44%	56%	44%	56%	33%	67%	21%	79%	22%	78%
mean	0.656		0.563		0.555		0.673		0.792		0.778	
std	0.475		0.499		0.497		0.469		0.406		0.441	
Male Primary Convener	5361	72%	227	72%	1641	70%	2028	72%	1286	74%	15	63%
	F	M	F	M	F	M	F	M	F	M	F	M
Invited Authors	1302	4059	86	141	542	1099	458	1570	186	1100	0	15
Invited Authors %	24%	76%	38%	62%	33%	67%	23%	77%	14%	86%	0%	100%
mean	0.757		0.621		0.670		0.774		0.855		1.00	
std	0.429		0.486		0.470		0.418		0.352		0.000	
$\chi 2$	**77.7**		0.883		**27.6**		**30.4**		**9.88**		3.64	
p-value	**<0.001**		0.347		**<0.001**		**<0.001**		**0.002**		0.057	

Supplementary Table 5. Analysis of Primary Convener Allocation of Oral Presentations by First Author Career Stage

	Total		*FA Student*		*FA Early Career*		*FA Mid-Career*		*FA Experienced*		*FA Retired*	
Total Abstracts	17553		5187		6519		3784		1713		44	
Female Primary Convener	4665	27%	1357	26%	1749	27%	1023	27%	448	26%	14	32%
	F	M	F	M	F	M	F	M	F	M	F	M
Assigned Authors	1733	2932	610	747	708	1041	307	716	90	358	2	12
Assigned Authors %	37%	63%	45%	55%	40%	60%	30%	70%	20%	80%	14%	86%
mean	0.629		0.550		0.595		0.700		0.799		0.857	
std	0.483		0.498		0.491		0.459		0.401		0.363	
Male Primary Convener	12888	73%	3830	74%	4770	73%	2761	73%	1265	74%	30	68%
	F	M	F	M	F	M	F	M	F	M	F	M
Assigned Authors	3769	9119	1521	2309	1519	3251	532	2229	152	1113	5	25
Assigned Authors %	29%	71%	40%	60%	32%	68%	19%	81%	12%	88%	17%	83%
mean	0.708		0.603		0.682		0.807		0.880		0.833	
std	0.455		0.489		0.466		0.394		0.325		0.379	
$\chi 2$	**99.5**		**11.4**		**42.4**		**49.9**		**17.8**		0.0404	
p-value	**<0.001**		**0.001**		**<0.001**		**<0.001**		**<0.001**		0.841	

Supplementary Table 6. Analysis of Primary Convener Allocation of Invited Authors by Primary Convener Career Stage

	PC Student		*PC Early Career*		*PC Mid-Career*		*PC Experienced*		*PC Retired*	
Total Abstracts	358		3068		2580		1217		67	
Female Primary Convener	164	46%	1069	35%	533	21%	257	21%	27	40%
	F	M	F	M	F	M	F	M	F	M
Invited Authors	61	103	358	711	184	349	91	166	8	19
Invited Authors %	37%	63%	33%	67%	35%	65%	35%	65%	30%	70%
mean	0.628		0.665		0.655		0.646		0.704	
std	0.485		0.472		0.476		0.479		0.465	
Male Primary Convener	194	54%	1999	65%	2047	79%	960	79%	40	60%
	F	M	F	M	F	M	F	M	F	M
Invited Authors	58	136	497	1502	509	1538	192	768	13	27
Invited Authors %	30%	70%	25%	75%	25%	75%	20%	80%	33%	68%
mean	0.701		0.751		0.751		0.800		0.675	
std	0.459		0.432		0.432		0.400		0.474	
$\chi 2$	2.13		**25.8**		**20.1**		**27.0**		0.0617	
p-value	0.144		**<0.001**		**<0.001**		**<0.001**		0.804	

Supplementary Table 7. Analysis of Primary Convener Allocation of Oral Presentations by Primary Convener Career Stage

	PC Student		*PC Early Career*		*PC Mid-Career*		*PC Experienced*		*PC Retired*	
Total Abstracts	785		7717		5921		2598		118	
Female Primary Convener	339	43%	2471	32%	1228	21%	503	19%	38	32%
	F	M	F	M	F	M	F	M	F	M
Assigned Authors	142	197	904	1567	447	781	197	306	12	26
Assigned Authors %	42%	58%	37%	63%	36%	64%	39%	61%	32%	68%
mean	0.581		0.634		0.636		0.608		0.684	
std	0.494		0.482		0.481		0.489		0.471	
Male Primary Convener	446	57%	5246	68%	4693	79%	2095	81%	80	68%
	F	M	F	M	F	M	F	M	F	M
Assigned Authors	122	324	1599	3647	1359	3334	565	1530	20	60
Assigned Authors %	27%	73%	30%	70%	29%	71%	27%	73%	25%	75%
mean	0.726		0.695		0.710		0.730		0.750	
std	0.446		0.460		0.454		0.444		0.436	
$\chi 2$	**18.2**		**28.6**		**25.4**		**29.1**		0.564	
p-value	**<0.001**		**<0.001**		**<0.001**		**<0.001**		0.453	

Supplementary Table 8. Analysis of Poster Only Selection by First Author by Career Stage

	Total		*Student*		*Early Career*		*Mid-Career*		*Experienced*		*Retired*	
Total Abstracts	43515		12660		15039		9982		4903		100	
	F	M	F	M	F	M	F	M	F	M	F	M
	13784	29731	5386	7274	5130	9909	2323	7659	735	4168	13	87
	32%	68%	43%	57%	34%	66%	23%	77%	15%	85%	13%	87%
Poster Only	4360	7807	2369	3016	1250	2496	515	1554	151	614	3	12
Poster Only %	32%	26%	44%	41%	24%	25%	22%	20%	21%	15%	23%	14%
mean	1.32	1.26	1.44	1.41	1.24	1.25	1.22	1.20	1.21	1.15	1.23	1.14
std	0.465	0.440	0.496	0.493	0.429	0.434	0.415	0.402	0.404	0.354	0.439	0.347
$\chi 2$	**135**		**8.05**		1.22		**3.83**		**16.0**		0.765	
p-value	**<0.001**		**0.005**		0.269		**0.050**		**<0.001**		0.382	
